# Expandable Graphite, Aluminum Diethylphospinate and Melamine Polyphosphate as Flame Retarding System in Glass Fiber-Reinforced PA6

**DOI:** 10.3390/polym14061263

**Published:** 2022-03-21

**Authors:** Florian Tomiak, Angelina Schoeffel, Klaus Rathberger, Dietmar Drummer

**Affiliations:** 1 Institute of Polymer Technology, Friedrich-Alexander-University Erlangen-Nürmberg, Am Weichselgarten 10, 91058 Erlangen, Germany; dietmar.drummer@fau.de; 2 Bavarian Polymer Institute, Friedrich-Alexander-University Erlangen-Nürnberg, Dr. Mack Strasse 77, 90762 Fuerth, Germany; 3 Georg H. Luh GmbH, Schoene Aussicht 39, 65396 Walluf, Germany; angelina.schoeffel@luh.de (A.S.); klaus.rathberger@luh.de (K.R.)

**Keywords:** nylon 6, PA6, aluminum diethylphosphinate, AlPi, melamine polyphosphate, MPP, expandable graphite, EG, flame retardant additive, synergistic effect

## Abstract

A flame retardant system based on expandable graphite (EG), aluminum diethylphosphinate (AlPI) and melamine polyphosphate (MPP) was investigated in glass fiber- (GF) reinforced polyamide 6 (PA6). Burning characteristics were evaluated via cone calorimeter, limiting oxygen index (LOI) and UL-94 tests. Thermogravimetric analysis (TGA) and coupled Fourier transform infrared spectroscopy (FTIR) was used to investigate the decomposition process as well as flame retardant modes of actions. Specifically, in the cone calorimeter tests, formulations containing EG showed excellent flame retardant properties for non-reinforced and reinforced PA6. The best performance was achieved for 25 wt.% glass fiber-reinforced PA6 containing solely 20 wt.% EG, corresponding to a measured pHRR of 134 kW/m^2^ and a total smoke production of 1.2 m^2^. Higher glass fiber contents of 45 wt.% (30 vol.%) revealed a lower char volume, which was attributed to both the limited space available for expansion and the sheer-induced reduction in particle size during processing. All of the reinforced PA6 formulations only achieved V2 classifications, but this was at low filling degrees (10 wt.%) for both net EG or EG/AlPi/MPP combinations. For GF-reinforced PA6 containing EG/AlPi/MPP mixtures, a synergistic effect was found to improve the oxygen index up to 30.6%.

## 1. Introduction

Polymers provide a wide range of unique properties. In relation to their weight, polymers have enormous mechanical stability, are electrically insulating and can easily be shaped into various geometries. By adding a wide range of possible additives and fillers, their properties can further be developed and designed to meet specific product requirements [1,2]. Applications that are substantially exposed to heat during usage often require a certain temperature and ignition resistance as well as low heat and smoke generation properties in the event of a fire. Specifically, polymers that are frequently used in many applications, predominantly due to a good cost–performance ratio, are comparatively easy to ignite and contribute significantly to heat and smoke generation. In order to meet stringent fire safety requirements and to reduce the risk of a potential fire hazard, flame retardancy properties must be adjusted, most commonly through the incorporation of flame retardant additives [3,4].

Polyamides are a widely used polymer grade for many technical applications. A wide range of flame retardant additives for polyamides have been studied, considering single and synergistic multi-material systems with and without fiber reinforcement. For PA6, systems based on aluminum diethylphosphinate (AlPi) [5,6], melamine polyphosphate (MPP) [5,7], red phosphorus and ammonium polyphosphate (APP) [8,9] have been found to be specifically effective. More recently, published studies on the use of a higher temperature stable EG grade in PA6 also showed promising flame retarding properties, specifically in cone calorimeter tests [10,11]. The performance of a flame retardant additive system does not only depend on the chemistry itself, but also on physical factors (e.g., fillers) which influence the exchange of recuperated heat and generated flammable gases over time. The most common way to improve the mechanical properties of engineering polymers is their reinforcement through glass fibers. Glass fibers provide an excellent cost–performance ratio. They are comparatively inexpensive, mechanically stable, non-electrically conductive, can easily be made surface modifiable and provide a wide range of properties through the variation of different fiber lengths [1]. Glass fiber-reinforced systems are known for a “wicking effect” [12]. When exposed to a flame, the surrounding polymer matrix gradually vaporizes, leaving partially protruding fibers. The low melt viscosity of decomposed polymer as well as the presence of a flame allow for a capillary force-driven polymer migration along the fiber surface, improving the fueling process.

The reaction mechanisms of AlPi have been extensively studied. When combined with PA6 and PA6.6, AlPi works both through vaporization and flame inhabitation effects as well as the forming of a phosphoric acid carbonized residue. Studies have shown that AlPi works specifically well as a flame retardant in non-reinforced PA6, improving limiting oxygen index (LOI) and UL-94 test results, but provides substantially weaker effects in reinforced systems [5]. MPP, as a single flame retardant additive in a PA6 matrix, does not exceed sufficient UL-94 classifications or LOI values due to an early depolymerization mode and is thus mostly used as a synergist [13]. For AlPi and MPP combinations, the mode of action is reported to change towards a predominantly mesophase reaction, creating less char residue while releasing phosphinic acid into the gas phase. AlPi/MPP combinations do not perform to a satisfactory degree in non-reinforced systems, though they have been reported to be effective in reinforced PA6 and PA6.6 [5,7,14]. 

As a non-phosphorus alternative, expandable graphite has recently been studied as a flame retardant additive in PA6 compounds [10,11]. When exposed to heat, an intercalated blowing agent expands the graphite flakes into a worm-like structure several hundred times the original volume. When incorporated into a polymer, the expanded structure forms a voluminous char, thermally isolating lower polymer layers and thus effectively reducing flame spread by lowering the pyrolysis gas flow feeding the flame. Due to its solely physical effect, expandable graphite can be used in various polymeric systems (e.g., PE [15,16,17,18], PP [19,20], PS [21], PVC [22], ABS [23], PA6 [24]). Please note that an appropriate temperature stability is mandatory for processing. The main mode of action has been reported to be independent of the polymeric system [25], whereas the flake size as well as the corresponding expansion volume are the most dominant factors effecting flame retardancy performance [26,27]. Accordingly, for EG used as a flame retardant additive in PA6, studies report especially strong performances for cone calorimeter tests at considerable low filling degrees of 15 wt.%. However, to achieve a UL-94 V0 classification, a filling degree of 25 wt.% was needed. A synergistic effect was reported for recipes containing EG/AlPi/MPP in non-reinforced PA6, resulting in a higher LOI value and a more sufficient V0 classification.

Within this paper, we provide insights into a multi-material flame retarding system for a glass fiber-reinforced polyamide 6 grade based on EG as the main ingredient, as well as an AlPi/MPP mixture as a minor component. The study corresponds to knowledge gained from non-reinforced PA6/EG/AlPi/MPP systems published in [10,11] and intends to research the same system in glass fiber-reinforced PA6. Fire performance was investigated by cone calorimetry, UL-94 and LOI fire testing. Thermogravimetrical analysis (TGA) and Fourier transformation infrared spectroscopy (FITR) gas analysis were conducted to gain insights into the decomposition behavior and the results, which are extensively discussed. Light microscopy was used to identify particle distributions and particle orientations within (non)-reinforced PA6 recipes. Residue analysis was performed by scanning electron microscopy (SEM) and energy dispersive X-ray spectroscopy (EDX). 

## 2. Materials and Methods

### 2.1. Materials and Preparation

A standard PA6 grade (PA6 B27E; density 1.13 g/cm^3^; MVR 130 cm^3^/10 min at 275 °C/5 kg) from BASF SE (Ludwigshafen, Germany), an expandable graphite GHL PX 95 HT 270 (70% > 50 mesh; pH 5–9) from LUH GmbH (Walluf, Germany) and two conventional phosphorus-based flame retardant additives, MP200 (density 1.85 g/cm^3^; pH 5.5–6.5; particle size d_50_: 10 µm; nitrogen 42.0–44.0%; phosphorus 12.0–14.0%; low water solubility) from BASF SE (Ludwigshafen, Germany) and Exolit OP 1230 (density 1.35 g/cm^3^; d_50_: 20–40 µm; phosphorus 23.3–24.0%, low water solubility) from Clariant AG (Muttenz, Switzerland) were used in this study. Furthermore, a surface modified-glass fiber, CS 7929 from Lanxess AG (Cologne, Germany), was used. Compounds were produced using a twin-screw extruder (co-rotating), DSE ZSE HP 27 from Leistritz GmbH (Nuremberg, Germany), operating two gravimetrical feeder units. Barrel temperatures were controlled between 230 °C–240 °C and the screw speed at 100 rounds per minute (rpm). The screw was configured with two downstream kneading zones. Non-reinforced formulations were compounded at 10 kg/h and reinforced formulations at 8 kg/h. For glass fiber-reinforced recipes, AlPi, MPP and EG were premixed using a universal powder mixer (200 u/min; 15 min). The strand was cooled by a water bath, chipped for the purpose of granulation and then dried. Samples were prepared by injection molding into 2 mm and 4 mm thick plates using an injection molding machine, Arburg Allrounder 370 V from Arburg GmbH + Co KG (Loßburg, Germany). Aggregate temperatures were controlled between 230 °C (die) and 220 °C, the injection speed was controlled at 60 mm/s and the mold temperature at 80 °C. Sample geometries were prepared by sawing and milling. The geometries prepared were as follows: Cone calorimeter samples 100 mm × 100 mm × 4 mm; LOI samples 125 mm × 10 mm × 2 mm; UL-94 125 mm × 13 mm × 2 mm. Samples were conditioned to be dry (70 °C vacuum) and humid (60% rel. humidity, 70 °C) until weight consistency.

### 2.2. Microscopy and Particle Distribution

Microscopy images were taken to identify filler distributions, changes in particle size and potential processing defects. Reflected light microscopy images were taken using an AxioImagerM2m from Zeiss AG (Oberkochen, Germany). Samples were prepared by embedding them in a transparent epoxy resin, with subsequent grounding and polishing. 

### 2.3. Thermal and Gas Analytics

Coupled thermogravimetric analysis (TGA) and Fourier transform infrared spectroscopy (FTIR) were conducted in a nitrogen atmosphere. A TGA STA F3 449 Jupiter from Netzsch (Selb, Germany) equipped with a high-speed furnace was used for the study. A steady heating rate of 20 K/min was used between 50 °C and 800 °C at a N2 flow rate of 70 mL/min. Due to high end temperatures, the sample carrier (TG-DSC) was equipped with aluminum oxide tilts. Sample weights were kept constant at 10 ± 1 mg (20 K/min). The onset temperature was defined as 99% residual mass. All tests were conducted three times, and averaged curves are presented. The FTIR unit Tensor 2 from Bruker Corp. (Billerica, Massachusetts, USA) was coupled by a 230 °C controlled transfer line. FTIR gas-cell temperatures were controlled at 200 °C; 32 scans were averaged. A 30 s measurement delay occurred between TGA and FTIR results, which corresponds to 10 °C. 

### 2.4. Fire Testing

To conduct the burning behavior, cone calorimeter (ISO 5660-1), UL-94 (DIN EN 60695-11-10/20) and limiting oxygen index (LOI) (DIN EN ISO 4589-2) tests were conducted. All testing devices were from Netzsch Taurus GmbH (Weimar, Germany). Cone calorimeter tests are considered to be one of the most important fire testing procedures, providing a key figure-based opportunity to evaluate material-specific burning characteristics throughout all burning stages. Key figures provided can be summarized as follows: heat release rate (HRR), total heat emitted (THE), mass loss rate (MLR), average mass loss rate (AMLR), smoke production rate (SPR), total smoke production (TSP), time of ignition (t_ign_) and average specific extinction area (ASEA). For tests conducted within this study, 100 mm × 100 mm × 4 mm samples were prepared and tested at a heater power of 50 kW/m^2^. All tests were repeated three times; averaged curves are presented and standard deviations given in the corresponding tables. Evaluation procedures followed the guidelines and recommendations of good scientific practice described in [28,29]. 

In order to evaluate the thermal barrier efficiency given by EG comparatively for (non-)reinforced PA6, temperature measurements were conducted during cone calorimeter testing. Three thermocouples were therefore installed at the lower specimen side. Measurements were repeated three times. Averaged curves are presented and discussed.

Limiting oxygen index (LOI, ASTM D-2863) tests are used to obtain comparative values to characterize the flammability properties of polymeric materials. The test setup provides a vertically attached rod-sample surrounded by a glass tube, limiting the test environment. While testing, the oxygen level is systematically varied and a 50 W flame applied six times for 5 s each in a candle-like setup. The highest value achieved represents the oxygen index (OI). Specimen geometries were 125 mm × 10 mm × 2 mm. 

UL-94 (DIN EN 60695-11) tests provide general information about the self-extinguishing properties of polymeric materials. Different test variations are defined within the norm, whereas we used UL-94 V test setups in this study. Test specimens were attached vertically and exposed twice 10 s to a 50 W flame. Unlike the candle-like test set-up in LOI testing, the flame exposure is applied from below. Results are classified into three categories: V0, V1 and V2. Classifications achieved are particularly dependent on burning times and burn-dripping. V0 classifications do not allow for burn dripping and enforce strong self-extinguishing behavior, whereas V2 classifications allow for burn dripping and longer burning times. Specimen tested in this paper were 125 mm × 13 mm × 2 mm. All tests were conducted in accordance to standards. 

### 2.5. Char Residue Analysis

Scanning electron microscopy (SEM) images were used to conduct burning residues after cone calorimeter testing. Therefore, a device from Zeiss AG (Oberkochen, Germany), the Ultra Plus, was used. Further analysis by energy dispersive X-ray spectroscopy (EDX) was applied on the residue in order to gain additional knowledge on chemical compositions. Samples were spattered with a platinum–palladium mixture.

## 3. Results

### 3.1. Microscopy

**Figure 1:**
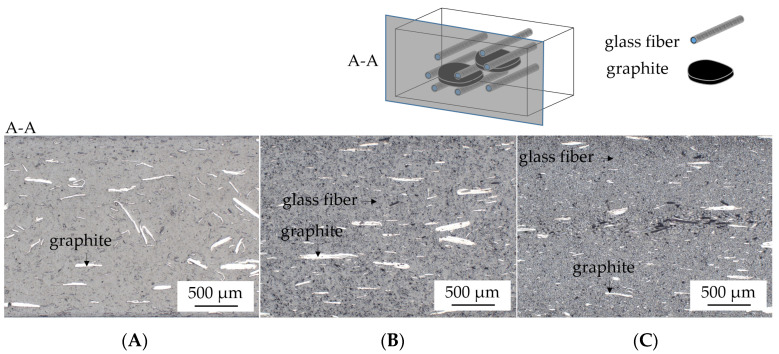
Microscopy images of (**A**) PA6 + 15 wt.% EG, (**B**) PA6 + 15 wt.% EG + 25 wt.% (15 vol.%) GF and (**C**) PA6 + 15 wt.% EG + 45 wt.% (30 vol.%).

Microscopy images were used to (1) ensure homogeneous particle/fiber distribution, (2) detect voids or other processing defects and (3) optically detect particle size distributions after processing. Representative images are presented in Figure 1. Microscopy images showed a homogeneous particle/fiber distribution. No optically detectable voids could be identified. However, in glass fiber-reinforced systems the graphite particles seemed to decrease in size. This indicates an increased sheer stress through increased particle–particle interactions, particularly evident in recipes containing 45 wt.% (30 vol.%) GF (Figure 1C). The particle sizes of EG are known to be proportionally related to the potential residue volume [20,30]. Since expected flame-retardant effects largely depend on the residue volume formed during combustion, a decrease in particle size can be assumed to reduce flame-inhibiting properties. These findings will be considered in the following discussion.

### 3.2. Thermal Analysis: TGA and DSC

**Figure 2:**
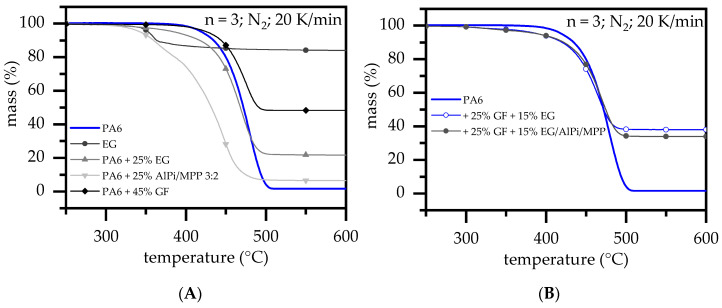
TGA analysis for a selection of formulations measured in a nitrogen atmosphere. (**A**) basic materials (**B**) multi-material formulations.

PA6 decomposed by a single mass loss step in TGA measurements, marking a decomposition onset T_99%_ at 394 °C, leaving almost no char residue, i.e., 1.6% (Figure 2A). The decomposition behavior of net PA6 is well known in the literature and will thus not be discussed extensively in the following [31,32,33,34,35,36]. EG particles are stacked graphene platelets that are intercalated by a blowing agent (mostly sulfur-containing acids) in between the graphene layers. Since graphite does not decompose under given conditions, a mass loss step found within TGA analysis can be assigned to the expansion reaction triggered by the blowing agent. The EG type presented has been fundamentally investigated in a recently published study [10]. TGA curves of up to 270 °C did not indicate any mass loss. A further temperature increase showed a steady decreasing characteristic between 270 °C and 340 °C, with the main mass loss step marked as a DTG peak at 357 °C (Table 1). Compared to net PA6, the decomposition process of PA6 containing 45 wt.% (30 vol.%) GF started at a slightly lower temperature of 381 °C. Mass loss rates occurring during decomposition appear to have been significantly lower due to the non-decomposable glass fiber residue present, whereas the overall mass loss characteristic did not imply changes within chemical reaction profiles. A residue of 48.3 ± 2 wt.% was identified, which corresponds well to a theoretical value of 46.6 wt.% (45 wt.% + 1.6 wt.%). 

For PA6/EG formulations, a recently published study found out that higher filling degrees tend to shift the decomposition onset (T_99%_) towards lower temperatures [10]. Since higher filling degrees converged regressively towards the decomposition onset temperature (T_99%_) of net EG, the increasing influence of the EG particles was concluded. No changes within the chemical decomposition pathway were found. [10] AlPi/MPP mixtures combined with EG were studied as a synergistic multi-material mixture in PA6 [11]. The general decomposition modes as well as the flame retardancy effects of PA6/AlPi/MPP mixtures have been described in detail in [5,7,11]. Net MPP is known to accelerate the decomposition process in PA6 due to an early de-polymerization mode. When combined with AlPi, in this case as the 3:2 AlPi/MPP mixture, the overall decomposition pathway changes. The reaction is known to act as combined mechanism, releasing some phosphoric acid (flame poisoning) a considerable amount of CO_2_ (dilution) and produces a low amounts of a phosphoric char residue. Thus, the residue is slightly higher than in the case of net PA6. Despite relatively lower concentrations of MPP, a large part of the decomposition takes place at low temperatures (T_99%_: 307 °C). Accordingly, an early de-polymerization mode also persists within AlPi/MPP mixtures, marking two decomposition peaks at 362 °C and 451 °C. When glass fiber-reinforced PA6 was combined with EG or EG/AlPi/MPP as a flame retardant multi-material system, the effects of individual additives and fillers are proportionally evident in the mass loss characteristics (Figure 2B). A low onset temperature remains for all formulations in a range of 295 °C to 306 °C, whereas the general decomposition characteristic evident in TGA curves did not change. The resulting residue thus exceeds expectation values. High GF weight fractions further decelerated the mass loss rate due to a non-disposable filler, leading to an increased non-combustible residue fraction. No significant differences were found for reinforced PA6 containing EG or EG/AlPi/MPP mixtures.

Considering melting characteristics, we found melting temperatures to decrease when higher GF fractions were present in the formulations (Table 1). A lower melting temperature in GF-modified PA6 could indicate a higher fraction of γ-phase crystals. The literature reports α- and γ-phase crystals to be predominant in PA6, which are mostly dependent on cooling conditions but can also be influenced by surface modifications. γ-phase crystals are characterized by somewhat lower melting temperatures, and the reported temperatures vary between 180 °C to 210 °C. Since melting temperatures decreased for the given material formulations with increasing GF filling degrees, we suggest a change in α-/γ-phase ratios. This might be attributed to the presence of a surface modification, which might have changed local crystallization kinetics in close proximity to glass fibers. However, further studies are needed to verify this proposal [37,38]. 

### 3.3. Evolved Gas Analysis: TGA-FTIR

**Figure 3:**
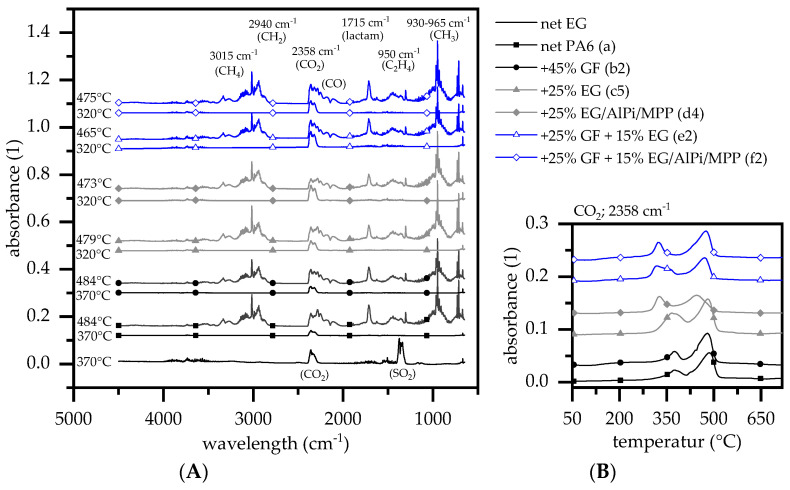
FTIR gas analytics plotted for a representative selection. (**A**) FTIR peak spectra for a representative sample selection; (**B**) CO_2_ trace at 2358 cm^−1^.

TGA-FTIR measurements were conducted at a heating rate of 20 K/min in a nitrogen atmosphere. The decomposition process of PA6 has been comprehensively studied and will thus not be discussed in detail [34,36,39]. PA6 is known to decompose mostly to its monomer caprolactam, followed by a further breakdown into subsequent products: CO, CO_2_, H_2_O, NH_3_ and HCN (Figure 3A) [40]. In a nitrogen atmosphere, the TGA measurements indicated a single step decomposition process (FTIR peak: 470 °C), whereas coupled FTIR analysis also revealed an additional, non gravimetrical decomposition step at lower temperatures releasing solely CO_2_ (FTIR peak: 370 °C). Since there is no mass loss step evident in the TGA curves, the decomposition processes were mainly located in the mesophase. This is generally assumed to originate from the hydrolytic scission of the C(O)-NH bond and the subsequent decomposition of carbon acid to –CO–, CO_2_ and water. [41,42] Higher temperatures accelerate the decomposition process of PA6, with gas phase products becoming increasingly dominant. The following gas phase products could be identified: caprolactam (1715 cm^−1^, fingerprint patterns 1305, 1352, 1361 and 2865 cm^−1^), ammonia (930, 965 cm^−1^), NH groups (3334 cm^−1^), CH2 groups (2940, 2865, 1440 cm^−1^), ethene (950 cm^−1^), methane (3015 cm^−1^, fingerprint pattern 1200–1400 cm^−1^), CO_2_ (2360, 671 cm^−1^), CO (2114, 2174 cm^−1^) and water (3853, 3400–4000 cm^−1^). This corresponds with decomposition products reported in the literature [43,44,45].

Compared to net PA6, glass fiber-reinforced PA6 revealed no differentiating gas phase products. Characteristic changes evident in the burning behavior of glass fiber-reinforced PA6 is therefore limited to physical mechanisms as well as a passive flame retarding effect arising through polymer substitution. Both effects limit the supply of pyrolysis gasses, decreasing the fire propagation speed, the calorific value and lowering the total heat released. Decomposition products evolving from PA6 containing EG have recently been studied and published [10,11]. When net EG decomposed, mainly CO_2_ and SO_2_ were evident in the gas phase. The TGA measurement revealed a mass loss of about 16 wt.% between 307 °C (T_99_) and 420 °C (∆-16%), whereas 80% was vaporized between 340–370 °C (DTG peak: 357 °C). In the PA6/EG formulations, the relative proportion of evaporated SO_2_ was negligible and could not be detected in the FTIR measurements. Net glass fiber-reinforced PA6 showed no changes in gas phase components. This is a little surprising, since GF is not known to interfere with decomposition processes. Physical effects, such as wicking, that might shift decomposition temperatures are not likely, since a heating rate of 20 K/min provided a rather homogenous heating throughout the milligram sample. Modes of fire retarding actions of AlPi and/or MPP have been studied as flame retardant additives within glass fiber-reinforced PA6 [5] and PA6.6 [7]. AlPi/MPP mixtures have been reported to act predominantly in the mesophase, forming a phosphoric char residue. Some aluminum phosphinate has also been reported to be a minor gas phase fraction (main traces of phosphinic acid at 855 cm^−1^ and phosphinate 1146 cm^−1^ [5]), though this could not be clearly identified within this study. 

### 3.4. Burning Behavior: Cone Calorimeter Test

**Figure 4:**
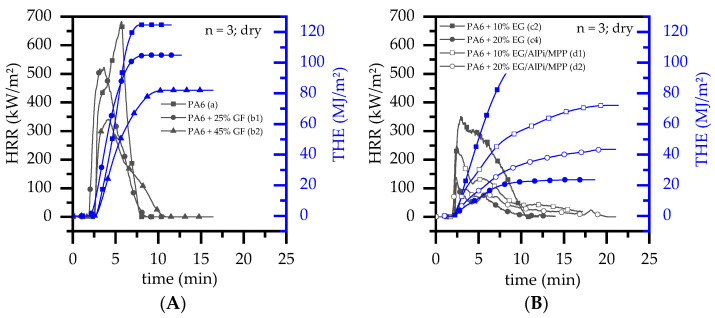
Representative selection of cone calorimeter (50 kW/m^2^) results displayed over time for (**A**) net PA6 and PA6 containing GF as reference and (**B**) PA6 containing EG or EG/AlPi/MPP. Results shown refer to dry-conditioned samples.

Net PA6 samples showed typical heat release characteristics in a cone calorimeter testing environment (Figure 4A). After ignition, the fire evolved rapidly towards high heat release rates, reaching a pHRR at 569 kW/m^2^ (wet)/708 kW/m^2^ (dry). The pHRR marked a turning point, where most of the fuel was already burned and a pyrolysis gas supply drastically declined until flame extinction. 

Specifically, during early burning stages, a thin carbon layer was formed for dry PA6 samples, which affected a delay in the ignition onset (t_ign_) as well as a subsequent increase in pHRR. No lasting char residue was formed. A heat capacity of 26 ± 1.1 J/g was calculated for dry PA6, which is in well accordance with values in the literature [46]. Melt dripping, which occurred during testing, is considered responsible for the deviation. When glass fibers were incorporated into PA6, the matrix gradually decomposed, leaving a growing amount of a thermally stable glass fiber network. This network influenced a variety of physical processes, whereas its effect can be observed and described by the following two characteristics:

(1) With ongoing fuel evaporation, an increasingly larger proportion of the glass fiber residue was exposed. The fiberglass network had a porous and easily gas-permeable characteristic, which offered little thermal protection, particularly during early burning stages. Instead, the residue promoted the mass transport of combustion gases from deeper material layers provided by the “wicking effect” and an enlarged surface area. Speaking of the characteristics recorded in the cone calorimeter testing environment, the pHRR relative to the ignition time was reached earlier than for net PA6. This development was also reflected in the mass loss rate (MLR). Whereas the MLR within the first 30 s after ignition for net PA6 was 2.9 ± 0.5 g/min, PA6 containing 25 wt.% (15 vol.%) and 45 wt.% (30 vol.%) glass fiber showed significantly higher rates at 3.8 ± 0.6 g/min (+30%) and 4.4 ± 0.4 g/min (+50%), respectively. (Please note: the given mass loss rates can be used only for comparative reasons. The calculated time range was chosen as the “best suitable range” for dry samples in order to representatively express early mass loss stages consolidated in one key figure.) With larger proportions of incorporated glass fibers, the relative amount of matrix polymer reduced, so that less burning material was available and a lower overall pHRR was achieved.
(2) The second effect that could be observed for solely glass fiber reinforced PA6 was an increasing amount of residue, acting to some extend as a physical barrier. Simultaneously, the quantity of potential fuels reduced, mutually supporting a lower pHRR as well as a lower average heat release rate (AHRE). This effect became more evident for PA6 containing higher glass fiber fractions, resulting in a more or less linear decline in important key figures.

EG acts exclusively by forming a voluminous, thermally stable residue and was recently studied as a flame retardant additive for PA6 [10,11]. The flame retarding effect has been reported to be mainly determined by the formation time as well as the intensity of the fully-developed residue volume, providing a long-term fire protection [10,26,27,30]. This corresponds to the findings of this study. Recipes containing higher EG filling degrees showed a lower heat generation over time when tested under cone calorimeter conditions (Figure 4B). This can be attributed to a larger number of particles located close to the surface, evolving a more voluminous and denser residue morphology, which in turn provided better thermal isolation.

Specifically, during early burning stages, decomposition mechanisms accelerated to form a high-yield pyrolysis gas stream and competed with a steadily reducing heat impact through the evolvement of a thermal barrier. Higher EG filling degrees provided better residue efficiency, lowering the pHRR for PA6 recipes containing 10 wt.% and 20 wt.% EG from 359 kW/m^2^ to 122 kW/m^2^ (dry). A similar trend can be identified for PA6/EG/AlPi/MPP recipes (Figure 4A). Compared to PA6 containing 10 wt.% EG, the pHRR of PA6 containing 10 wt.% EG/AlPi/MPP showed a significant lower pHRR of 204 kW/m^2^ (↓ 38%), but a slightly higher pHRR for PA6 containing 20 wt.% EG/AlPi/MPP of 126 kW/m^2^ (↑ 17%). This indicates the strong impact of the AlPi/MPP synergism reported in [11], providing a visible effect for low EG filling degrees where the barrier effect is less sufficient. 

**Figure 5:**
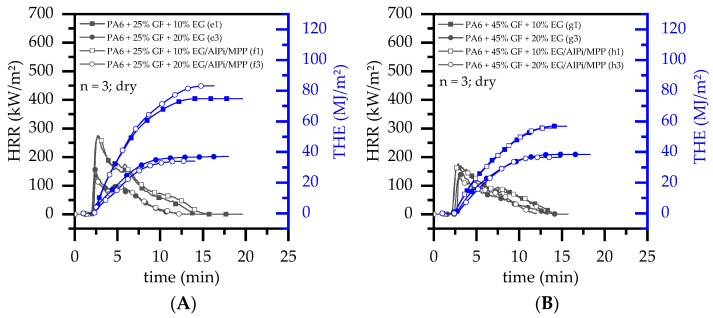
Representative selection of cone calorimeter (50 kW/m^2^) results displayed over time for (**A**) net PA6 and PA6 containing 25 wt.% and (**B**) 45 wt.% GF (15 vol.% and 30 vol.%) as well as 10 wt.% or 20 wt.% EG or EG/AlPi/MPP. Results shown refer to dry conditioned samples.

When glass fibers were added into the system, further PA6 fractions were substituted by a non-decomposable filler (Figure 5). Although the pHRR and the THE reduced as the EG filling degree increased, the flame inhibition effects measured were significantly lower than evident for non-reinforced systems. Accordingly, while non-reinforced PA6 containing 20 wt.% EG achieved a pHRR of 122 kW/m^2^ and a THE of 23 MJ/m^2^ (dry), comparative values for reinforced systems containing 20 wt.% EG and 25 wt.% GF were 179 kW/m^2^/37 MJ and systems containing 45 wt.% GF were 157 kW/m^2^/38 MJ/m^2^. Similar trends were identified for PA6/GF/EG/AlPi/MPP formulations. 

**Figure 6:**
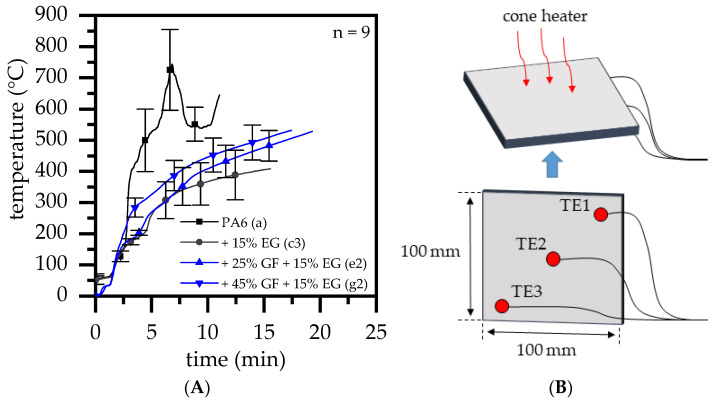
(**A**) Temperature measurements at the lower sample side during cone calorimeter testing in order to identify thermal insulation properties of non-reinforced and reinforced formulations. (**B**) Illustration of thermocouple positioning.

In order to comparatively evaluate the efficiency of the thermal barrier provided by EG for (non-)reinforced PA6, temperature measurements were conducted during cone calorimeter testing. Three thermocouples were therefore installed at the lower specimen side, as shown in Figure 6B. Measurements were repeated three times; averaged curves are presented in Figure 6A. The absence of a thermally protective residue for net PA6 provided an unrestrained heat impact, resulting in a rapid temperature buildup though the entire sample. An averaged maximum temperature of 740 ± 100 °C was measured, marking the coincident time of occurrence with the measured pHRR. EG reduced the heat input, as expected. Enabled by an increasing volume of residue, heating rates reduced over time, evident in a regressive temperature curve characteristic. In contrast, glass fiber-reinforced systems exhibited significantly higher end temperatures at the same EG filling level. Formulations containing 25 wt.% GF initially showed a congruent curve, matching the non-reinforced reference. After around seven minutes the non-reinforced formulation switched to a merely regressive characteristic, whereas temperatures measured for reinforced formulations kept rising. This indicates that the expansion behavior was initially identical to that of unreinforced systems, but that it ultimately resulted in a lower residual volume. A different situation applied to formulations containing 45 wt.% GF. The temperature rise separated from its reference course at an earlier point in time, exhibiting a generally higher temperature level. The development of a higher temperature during early burning stages, as well as a significantly higher end temperature compared to the reference, indicates that the development of the residue proceeded more slowly and resulted in a generally lower residue volume. We assume that there are two possible reasons for this:

(1) Results generated by cone calorimeter tests are particularly sensitive to effective residue formation. Compared to non-reinforced PA6 systems containing EG, high GF fractions seemed to reduce the resulting expansion volume as well as the built up speed. We assume two complementary effects to be responsible: (a) a spatially restricted freedom arising from the GF network blocking EG expansion and (b) process-induced sheering, which caused a reduction the size of EG particles, as seen in Figure 1, and thus a decrease in the potential blowing volume.
(2) As reported for net PA6/GF formulations, GF residue networks provide an accelerated combustion process, via wicking, as well as a gas permeable residual system. Pyrolysis gases can thus be provided from lower layers, leading to an improved fuel feed flow towards the flame. As a result, higher burning rates accelerate the development of heat and thus result in higher measured temperatures.

**Figure 7:**
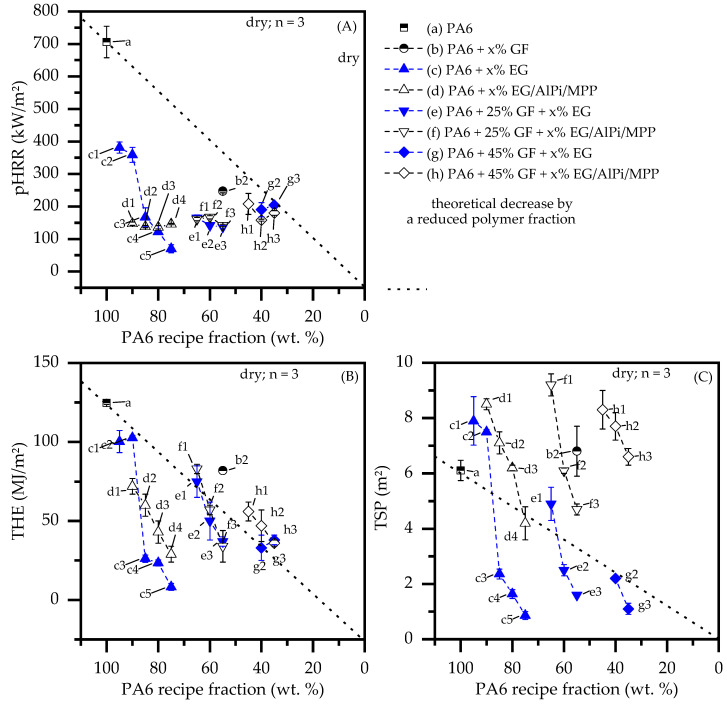
Comparison of major cone calorimeter (50 kW/m^2^) key figures applied over the PA6 matrix fraction. (**A**) peak heat release rate (pHRR); (**B**) total heat emitted (THE); (**C**) total smoke production (TSP).

The incorporation of non-combustible fillers into a polymer matrix also provides a passive flame retardant effect through substitution of combustible fuel. In order to evaluate the net flame retardant performance of all formulations, a selection of important key figures determined by cone calorimeter tests were plotted against the revealing weight percent fraction of PA6 (Figure 7). The dotted line in the diagram provides a theoretical indication of a linear decrease relative to the lower polymer fraction. The linear indication does not necessarily represent a realistic key figure development, but it does provide a solid basis for comparison. The results provided refer to dry samples. The conditioned samples show an identical pattern, with slightly lower values due to an endotherm cooling effect. Corresponding values are presented in Table 2. 

The PA6/GF formulations achieved a slightly higher pHRR than the linear expectation value, but they remained within the standard deviation corridor (Figure 7A). A decrease in the pHRR is therefore mainly attributable to a fuel substitution effect, whereas a slightly higher tendency can be explained by wicking and an increased surface exposure, as described earlier. The non-reinforced formulations containing EG and EG/AlPi/MPP outperformed all the other formulations tested. They achieved the lowest pHRR and THE values measured despite a relatively high polymer fraction (Figure 7A,B). As described earlier, glass fiber reinforced systems lose flame inhibition efficiency relative to the polymeric substitution, specifically for higher GF filling degrees. Since the expansion volume decreases with higher GF contents, the predominant contribution of flame retarding effects is achieved a via polymeric substitution. The THE key figure did not improve for reinforced systems. Despite the significantly lower polymer contents of GF-reinforced formulations, all the key figures measured were within the linear expectation range. 

The total smoke production is another important key figure to evaluate. Very low TSP values were measured for non-reinforced and reinforced PA6 formulations containing solely EG as a flame retarding additive. In contrast, formulations containing AlPi/MPP as an additional ingredient showed higher smoke production tendencies. This is not surprising, since AlPi/MPP interferes with the decomposition processes of PA6, resulting in a lower combustion efficiency and thus higher smoke formation. However, non-reinforced PA6 containing EG/AlPi/MPP resulted in lower TSP values equal to or lower that the linear expectation value, which can be attributed to a generally low burning rate as well as a higher non-decomposed polymer fraction. 

### 3.5. Burning Behavior: UL-94 and LOI

**Figure 8:**
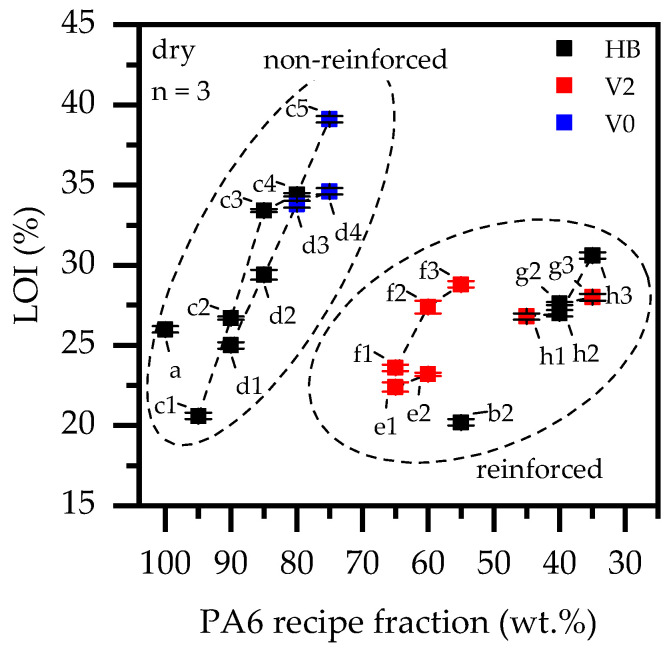
UL-94 and LOI test results for 2 mm thick samples.

In UL-94 V fire tests, rapid extinguishing properties are rated favorably, even when achieved by burn-dripping or burn-breaking. PA6 is characterized by a very low melt viscosity, which tends to drip when exposed to flame. In a UL-94 V test environment, PA6 exhibited severe flame dripping with subsequent self-extinction and therefore achieved a V2 classification. The corresponding oxygen index was 26% (Figure 8, Table 3). When fillers are added, PA6 generally loses its ability to drip. In a UL-94 testing environment, glass fiber reinforced PA6 showed improved melt stability and burned through completely after initial ignition, resulting in no classification. LOI results showed a similar behavior. No melt flow could be observed leaving the burning area. Accordingly, the LOI measured was 20%. 

As expected, the self-extinguishing properties of PA6 improved significantly with the addition of EG and achieved a V0 classification when 25 wt.% were added. A corresponding LOI value of 39% was found. Burn breaking occurred for lower filling degrees, which only led to self-extinguishment in the case of 20 wt.% EG. The fire behavior and efficiency of PA6/EG formulations has been studied in detail in [10] and is therefore not discussed further here. 

A combination of EG and AlPi/MPP (3:2) has been shown to act synergistically in non-reinforced PA6 formulations [11]. Not surprisingly, when EG/AlPi/MPP was used as a synergistic multi-material flame retardant system, modified PA6 achieved a V0 classification for slightly lower filling degrees of 20 wt%. No burn-dripping occurred, accompanied by almost instantaneous extinction behavior, which was also evident in high LOI values of 35% and 34% for 25 wt.% and 20 wt.% formulations. AlPi/MPP combinations were reported to act mainly via CO_2_ evaporation, a mesophase mechanism leading to residue formation by aluminum phosphate and phosphinic acid and some gas-phase flame effects by phosphoric acid [5,7,11]. 

When EG was added into glass fiber-reinforced PA6, the highest UL-94 classification achieved was V2 (Figure 8). All samples showed burn-breaking characteristics, whereas the char residue expansion provided by EG particles seemed to develop more slowly than that observed for non-reinforced PA6. Even though an isolating effect was evident due to steadily decreasing burning times when higher filling degrees were applied, not enough residue was formed to provide quick self-extinguishment or non-burn dripping behavior. 

Nevertheless, for PA6 containing 25 wt.% (15 vol.%) GF as well as either EG or EG/AlPi/MPP as flame retardant additives, all formulations achieved V2 classifications, even for low filling degrees. Despite the same UL-94 classification, PA6/GF formulations containing EG/AlPi/MPP tended to achieve a slightly higher LOI value. This implies the presence of a synergistic effect, though not strong enough to improve the UL-94 classification. Higher GF contents of 45 wt.% (30 vol.%) further reduced the flame inhibition performance, providing lower UL-94 classifications while maintaining an LOI value measured in the range between 25% and 30%.

As discovered for cone calorimeter tests, we attribute the lower flame inhibition properties discovered for reinforced PA6 to (a) a limitated expansion provided by presence of a glass fiber network, (b) a sheer-induced reduction in particle size via production and (c) that fact that wicking increased surface areas, providing a better fuel supply. Lower expansion volumes and a slower residue build up were optically evident in both tests. All testing results for LOI and UL-94 are listed in Table 3.

### 3.6. Char Residue Analysis: SEM and EDX Analysis

**Figure 9:**
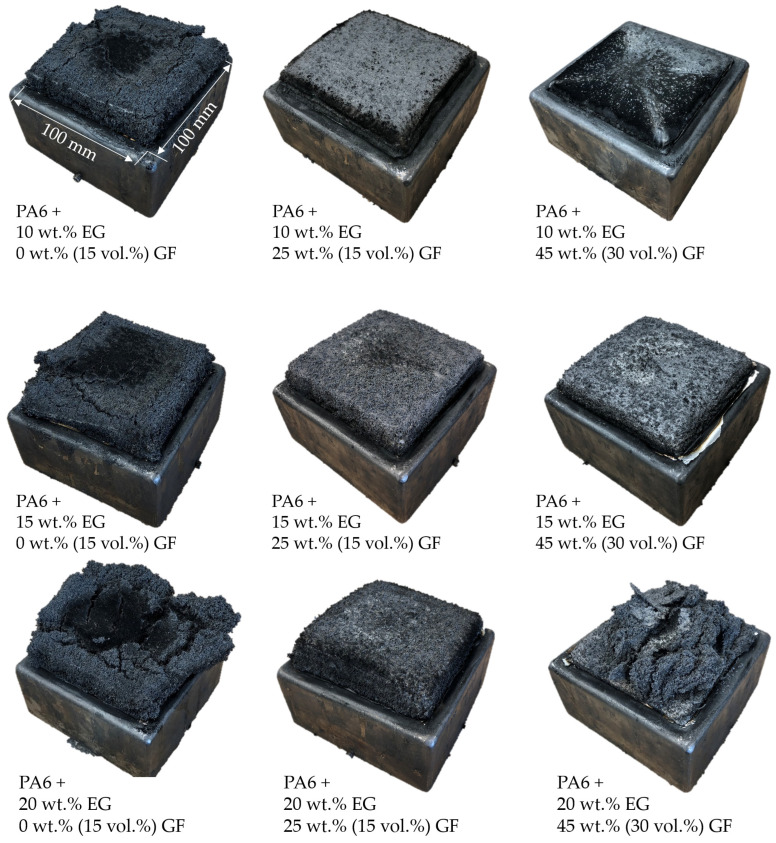
Decreasing residue volumes for higher GF contents in PA6/EG/GF formulations. Cone calorimeter residues after testing at 50 kW/m^2^, presented for non- reinforced and reinforced PA6 at different EG and GF filling degrees.

Regardless of the specific formulation, an increased residue volume for higher EG filling degrees was evident (Figure 9). PA6/EG as well as PA6/EG/AlPi/MPP formulations achieved the highest residue volumes (Figure 9), but had lower residue stabilities than GF-reinforced formulations. The presence of GF determined an overall decrease in the volume of residue and was particularly evident in systems containing a high GF fraction. Whereas formulations containing 25 wt.% GF showed little overall limitations in residue volume, representing a rather balanced ratio between expansion and residue stability, formulations containing 45 wt.% GF appeared to significantly limit the expansion behavior. This implies countervailing forces provided by the inflexible glass fiber network acting against the EG expansion process. Thus, PA6 containing 10 wt.% EG and 45 wt.% GF resulted in no remarkable amount of residue (Figure 9). Identical formulations containing higher EG fractions even showed considerable surface breakage, leaving a destructed residue with low flame inhibition properties (Figure 9). 

**Figure 10:**
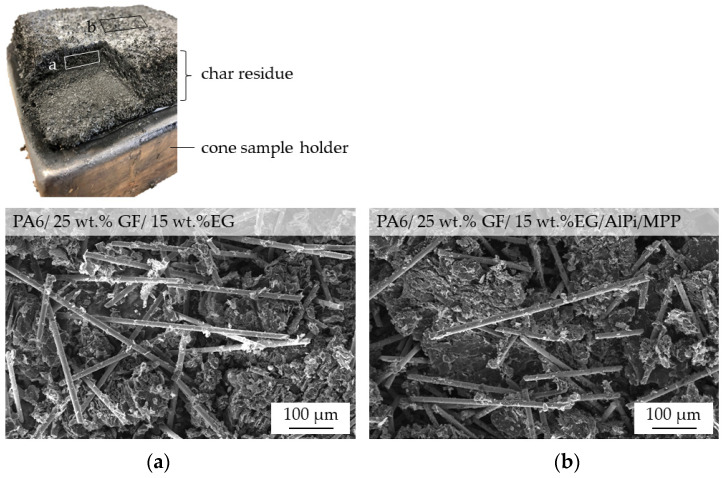
REM image of char residue after cone calorimeter testing: (**a**) PA6 + 25 wt.% (15 vol.%) GF + EG, (**b**) PA6 + 25 wt.% (15 vol.%) GF + EG/(AlPi/MPP 3:2).

SEM images of PA6/GF/EG and PA6/GF/EG/AlPi/MPP residues optically showed no significant differences in its appearance (Figure 10). The expanded graphite particles and glass fibers formed an entangled, open-pored network, which was responsible for the increase in stability compared to net PA6/EG or PA6/EG/AlPi/MPP formulations. Some connections between graphite particles and glass fibers were evident. However, since these seemed to exist only partially, the increase in stability was caused both by physical entanglement and chemical bonding.

For residues in between the glass fibers, a different atomic composition could be identified by SEM-EDX analysis. Besides C, O and Si atoms, the analysis revealed a considerable intensity of phosphorus and aluminum atoms present in PA6/GF/EG/AlPi/MPP residues. This indicates aluminum phosphoric char residue fractions and corresponds to findings reported for PA6/GF/AlPi/MPP formulations [5,7].

## 4. Discussion

Non-reinforced and reinforced PA6 formulations were investigated in various combinations with EG, AlPi and MPP as flame retardant additives. The study was intended to investigate changes in the burning behavior provided by a glass fiber (GF) network as well as to determine whether EG/AlPi/MPP additives exhibit synergistic flame retarding effects in glass fiber-reinforced PA6. 

Expandable graphite provides excellent flame retarding properties, which are particularly effective in the long term to reduce fire spread. The fire protection effect, as reported for PA6 [10] as well as for other polymeric material systems (e.g., PE [15,16,17,18], PP [19,20], PS [21], PVC [22], ABS [23], PA6 [24]), is based solely on the physical formation of a thermally stable, voluminous residue. Since the residue initially starts to form when exposed to heat, an efficient flame retardancy effect initially becomes effective with a certain time delay. Flammability tests such as LOI tests or self-extinguishing tests such as UL94 tests examine initial material properties at a very early stage of heat exposure. Only little time is given for the formation of an effective residue, and so sufficient values and classifications were only reached at high filling degrees. AlPi/MPP combinations have been reported to work synergistically in non-reinforced PA6 for EG/AlPi/MPP [11] formulations as well as in reinforced PA6 for AlPi/MPP formulations [5]. This corresponds to the findings of this study, where we found PA6 containing 20 wt.% of an EG/AlPi/MPP mixture to achieve a V0 classification in 2 mm thick samples. Lower filling degrees failed the test. 

For glass reinforced PA6, we found EG or EG/AlPi/MPP to be less efficient. Although reinforced formulations still performed well in cone calorimeter tests, pHRR and THE key figures deteriorated considerably when compared to non-reinforced systems. Glass fiber residue reduced the available space and, due to its inflexible characteristics, restricted the expansion process of EG. This could be observed in a slower residual built up process shortly after ignition and subsequent volume development to form an efficient thermal barrier. For high GF filler contents of 45 wt.% (30 vol.%) in particular, a limited expansion volume was clearly evident. PA6 containing 10 wt.% and 45 wt.% (30 vol.%) GF resulted in nearly no expansion volume, whereas PA6 containing 15 wt.% and 45 wt.% (30 vol.%) GF resulted in substantially less expansion volume compared to non-reinforced systems. Both exhibited a very dense and compact residue characteristic, withstanding an impressive amount of manual applied force. The intensity of opposing forces between a residual glass fiber network as well as the expansion mechanism of EG became particularly clear when examining formulations containing 20 wt.% EG and 45 wt.% (30 vol.% GF). During cone calorimeter testing, the expansion pressure of EG particles exceeded the local restraining forces provided by the GF network. As a result, the surface burst open, exposing lower polymer layers and thus drastically reducing the barrier effect. This phenomenon first occurred during early burning stages and subsequently intensified. 

Other influencing factors were identified, such as processing and increased sheer stress. High filling degrees reduce the viscosity of polymeric systems due to a lower matrix content and increased particle–particle and particle–matrix interactions. EG particles consist of stacked graphene platelets whose bonding perpendicular to the stacking direction is based on Van-der-Waals forces. These tend to cave under increased shear stress, reducing the stack size and thus lowering the blowing potential. We found solid evidence that, particularly for higher GF filling degrees of 45 wt.% (30 vol.%), processing-induced sheer stress resulted in a decrease in the size of EG particle. This was complementary to restrictions provided by the glass fiber network, resulting in a further decrease in the blowing volume. 

Besides having restrictive characteristics, glass fibers were found to propagate decomposition and pyrolysis. Compared to net PA6, the early burning stages just after ignition indicated a substantial increase in the mass loss rate (MLR) when higher GF fractions were present (25 wt.% GF: +30%; 45 wt.%: +50%). Since pyrolysis gases during this initial stage are generated almost exclusively in the upper specimen layer, we attributed this effect to “wicking” as well as an increased surface area. Partially exposed glass fibers improve the heat input, provide a better thermal conductivity along glass fibers and support an upward polymer flow driven by capillary forces. It is likely that wicking and the increased surface area also contribute during advanced burning stages. However, this study cannot provide concrete evidence to underline this explanation. 

The highest classification achieved in UL-94 tests for reinforced recipes was V2. Even though we found evidence that AlPi/MPP contributes synergistically in LOI tests, the mechanism was not strong enough to increase the UL-94 classification to V0. Thus, a sufficient effect for EG/AlPi/MPP systems as detected for non-reinforced formulations could not be found for GF reinforcement. UL-94 tests evaluate self-extinguishing properties during early burning stages. As described earlier, glass fiber reinforcement tends to decrease the speed of residual build up, delaying the formation of a protective thermal barrier. However, V2 classifications were already reached for filling degrees as low as 10 wt.%. Char residue characteristics for GF-reinforced systems containing EG particles where superior in matters of stability, withstanding impressive manual impact forces. This might be of particular importance for some applications that require long term residue stability even under certain physical stress while providing good long term properties, inhibiting the spread of flames. 

## 5. Conclusions

The flame inhabitation properties of expandable graphite were investigated for glass fiber-reinforced systems based on PA6. The burning behavior was analyzed by cone calorimeter, UL-94 and LOI burning tests. TGA-FTIR analytics were used in order to gain insight into the decomposition process; char residues were investigated by microscopy, REM and EDX analysis. All the essential findings can be concluded as follows:

- Glass fiber reinforcement changes the expansion behavior of EG particles and thus reduces any flame retarding effects provided. Substantially higher filling degrees tend to substantially decrease resulting blowing volumes. We assumed two reasons for this: (1) glass fibers provide a mechanically stable network in burning residues, limiting the space for and the expansion possibilities of EG.; (2) increased sheer stress via particle–particle or particle –fiber interactions reduces the size of EG particle, thus reducing the blowing potential.
- A synergistic flame retarding effect for EG/AlPi/MPP recipes, as reported for non-reinforced PA6 [11], was found to be not sufficient. The highest UL-94 classification achieved for reinforced PA6 formulations was V2. All formulations achieved a V2 classification for filling degrees as low as 10 wt.% EG or EG/AlPi/MPP.
- The char residue characteristics for GF-reinforced systems containing EG particles were superior in matters of stability, withstanding impressive manual impact forces. This might be of particular importance for some applications that require long term residue stability even under certain physical stress while providing good long term properties, inhibiting the spread of flames.
- As found for non-reinforced systems, EG-modified PA6 formulations showed very low smoke production. Due to an additional gas phase action, EG/AlPi/MPP resulted in slightly higher smoke production.

## Figures and Tables

**Table 1:** TGA and DSC measurement results summary.

					DSC	TGA		
	PA6 wt.%	GF wt.%	EG wt.%	AlPi/MPP (3:2) wt.%	T_m_ Onset/Peak	T_99%_ Onset °C	DTG-Peak °C/min	Residue%
a	100				218 ± 1/232 ± 0	394 ± 1	479 ± 2	1.6 ± 0
			100		-	307 ± 1	357 ± 2	84.0 ± 0
b2	65	45			211 ± 1/225 ± 1	381 ± 2	474 ± 2	48.3 ± 2
	75			25		307 ± 5	362; 451 ± 2	6.3 ± 1
c3	85		15		220 ± 1/232 ± 0	332 ± 2	476 ± 2	13.1 ± 1
c5	75		25		220 ± 1/232 ± 0	316 ± 2	468 ± 2	21.7 ± 1
d2	85		12	3	219 ± 1/	314 ± 3	467 ± 2	14.2 ± 1
	75		20	5	219 ± 1/	306 ± 2	464 ± 2	19.3 ± 1
e2	60	25	15		211 ± 2/225 ± 1	306 ± 3	465 ± 2	38.0 ± 2
f2	60	25	12	3	211 ± 1/225 ± 1	310 ± 3	470 ± 2	33.9 ± 2
g2	40	45	15		206 ± 1/221 ± 1	302 ± 3	463 ± 2	59.0 ± 2
h2	40	45	12	3	206 ± 2/222 ± 1	295 ± 4	466 ± 2	57.5 ± 2

**Table 2:** Summary of cone calorimeter fire testing results; 50 kW/m^2^.

	PA6 wt.%	GF wt.%	EG wt.%	AlPi/MPP (3:2) wt.%	t_ign_ s	pHRR kW/m^2^	THE MJ/m^2^	MAHRE kW/m^2^	TSP m^2^
	humid								
a	100	0			54 ± 2	569 ± 21	100 ± 0	257 ± 21	5.6 ± 0.7
b1	75	25			47 ± 2	561 ± 13	110 ± 4	264 ± 8	6.0 ± 0.1
b2	65	45			55 ± 2	369 ± 4	77 ± 4	149 ± 10	4.5 ± 0.5
c1	95		5		42 ± 3	353 ± 5	88 ± 0	163 ± 2	7.0 ± 0.1
c2	90		10		45 ± 5	373 ± 10	97 ± 2	184 ± 8	6.6 ± 0.6
c3	85		15		51 ± 4	133 ± 5	23 ± 2	58 ± 2	1.7 ± 0.4
c4	80		20		51 ± 2	88 ± 5	15 ± 1	40 ± 4	0.9 ± 0.1
c5	75		25		55 ± 4	62 ± 10	8 ± 2	20 ± 5	0.4 ± 0.1
d1	90		8	2	41 ± 8	220 ± 9	62 ± 2	89 ± 9	6.2 ± 0.6
d2	85		12	3	48 ± 2	189 ± 29	46 ± 6	66 ± 2	5.6 ± 0.4
d3	80		16	4	49 ± 3	140 ± 15	32 ± 4	52 ± 2	4.1 ± 0.4
d4	75		20	5	52 ± 3	75 ± 5	14 ± 2	31 ± 4	2.4 ± 0.2
e1	65	25	10		47 ± 2	249 ± 7	74 ± 8	128 ± 2	3.4 ± 0.2
e2	60	25	15		49 ± 1	172 ± 2	45 ± 5	84 ± 6	2.0 ± 0.2
e3	55	25	20		60 ± 1	134 ± 7	31 ± 5	66 ± 6	1.2 ± 0.2
f1	65	25	8	2	54 ± 2	270 ± 10	73 ± 5	116 ± 3	6.7 ± 0.6
f2	60	25	12	3	56 ± 2	191 ± 15	46 ± 3	84 ± 3	5.1 ± 0.4
f3	55	25	16	4	56 ± 4	155 ± 6	35 ± 5	64 ± 3	4.3 ± 0.2
g2	40	45	15		68 ± 3	145 ± 11	45 ±1	76 ± 5	1.9 ± 0.1
g3	35	45	20		91 ± 4	150 ± 35	23 ± 4	49 ± 11	0.6 ± 0.2
h1	45	45	8	2	61 ± 7	172 ± 6	56 ± 11	80 ±6	5.4 ± 0.4
h2	40	45	12	3	71 ± 6	158 ± 5	54 ± 0	78 ± 3	6.5 ± 0.2
h3	35	45	16	4	80 ± 7	109 ± 12	33 ± 2	56 ± 4	4.1 ± 0.2
	dry								
a	100	0			74 ± 4	708 ± 45	134 ± 3	328 ± 12	7.4 ± 0.2
b1	75	25			40 ± 1	550 ± 4	105 ± 4	258 ± 5	6.9 ± 0.1
b2	65	45			71 ± 4	362 ± 22	82 ± 0	156 ± 8	6.8 ± 0.9
c1	95		5		52 ± 4	381 ± 17	100 ± 7	181 ± 23	7.9 ± 0.9
c2	90		10		54 ± 1	359 ± 23	102 ± 2	191 ± 3	7.5 ± 0.1
c3	85		15		55 ± 3	167 ± 29	26 ± 2	49 ± 1	2.4 ± 0.2
c4	80		20		60 ± 1	122 ± 3	23 ± 2	44 ± 4	1.6 ± 0.2
c5	75		25		64 ± 2	70 ± 12	8 ± 2	21 ± 6	0.9 ± 0.1
d1	90		8	2	49 ± 2	204 ± 118	72 ± 5	106 ± 11	8.5 ± 0.2
d2	85		12	3	51 ± 3	160 ± 94	60 ± 7	78 ± 11	7.1 ± 0.4
d3	80		16	4	50 ± 5	126 ± 73	43 ± 7	61 ± 11	6.2 ± 0.1
d4	75		20	5	59 ± 2	137 ± 8	29 ± 5	49 ± 7	4.2 ± 0.6
e1	65	25	10		53 ± 2	304 ± 13	75 ± 10	125 ± 16	4.9 ± 0.6
e2	60	25	15		51 ± 3	179 ± 105	50 ± 12	66 ± 39	2.5 ± 0.2
e3	55	25	20		50 ± 4	179 ± 8	37 ± 3	68 ± 2	1.6 ± 0.0
f1	65	25	8	2	59 ± 5	223 ± 129	83 ± 3	129 ± 6	9.2 ± 0.4
f2	60	25	12	3	57 ± 3	201 ± 3	57 ± 3	94 ± 6	6.1 ± 0.1
f3	55	25	16	4	59 ± 2	152 ± 35	34 ± 10	62 ± 14	4.7 ± 0.2
g1	45	45	10		66 ± 7	183 ± 10	57 ± 2	85 ± 2	2.8 ± 0.1
g2	40	45	15		66 ± 2	89 ± 53	33 ± 8	43 ± 26	2.2 ± 0.1
g3	35	45	20		73 ± 1	157 ± 14	38 ± 3	62 ± 3	1.1 ± 0.2
h1	45	45	8	2	63 ± 6	197 ± 10	56 ± 6	84 ± 10	8.3 ± 0.7
h2	40	45	12	3	72 ± 4	180 ± 23	47 ± 10	76 ± 15	7.7 ± 0.5
h3	35	45	16	4	90 ± 2	145 ± 8	36 ± 1.9	61 ± 2	6.6 ± 0.3

**Table 3:** Summary of UL-94 and LOI testing results.

	PA6 wt.%	GF wt.%	EG wt.%	AlPi/MPP (3:2) wt.%	UL-94 (2 mm)	t1 s	t2 s	C_ign_ *	LOI (2 mm)%
	dry								
a	100	0			V2	3 ± 2	4 ± 2	yes	26.0 ± 0.2
b1	75	25			HB	55 ± 6	-	yes	-
b2	65	45			HB	47 ± 3	-	yes	20.2 ± 0.2
c1	95		5		V2	2 ± 1	7 ± 2	yes	20.6 ± 0.2
c2	90		10		HB	4 ± 1	8 ± 3	yes	26.7 ± 0.1
c3	85		15		HB	7 ± 1	7 ± 2	yes	33.4 ± 0.1
c4	80		20		V2	5 ± 2	6 ± 2	yes	34.4 ± 0.1
c5	75		25		V0	2 ± 1	2 ± 1	no	39.1 ± 0.2
d1	90		8	2	HB	31 ± 10	12 ± 12	yes	25.0 ± 0.2
d2	85		12	3	HB	9 ± 4	12 ± 7	yes	29.4 ± 0.3
d3	80		16	4	V0	0 ± 0	0 ± 0	no	33.8 ± 0.2
d4	75		20	5	V0	0 ± 0	0 ± 0	no	34.6 ± 0.2
e1	65	25	10		V2	16 ± 1	11 ± 10	yes	22.4 ± 0.3
e2	60	25	15		V2	14 ± 2	7 ± 5	yes	23.2 ± 0.1
e3	55	25	20		V2	10 ± 2	8 ± 3	yes	-
f1	65	25	8	2	V2	17 ± 2	4 ± 2	yes	23.6 ± 0.2
f2	60	25	12	3	V2	15 ± 6	9 ± 4	yes	27.4 ± 0.4
f3	55	25	16	4	V2	6 ± 7	7 ± 3	yes	28.8 ± 0.2
g1	45	45	10		-	-	-	-	-
g2	40	45	15		HB	23.04	13 ± 10	yes	27.6 ± 0.1
g3	35	45	20		V2	19 ± 3	4 ± 2	yes	-
h1	45	45	8	2	V2	13 ± 8	12 ± 8	yes	26.8 ± 0.2
h2	40	45	12	3	HB	22 ± 15	17 ± 13	yes	27.0 ± 0.2
h3	35	45	16	4	HB	17 ± 20	6 ± 6	yes	30.6 ± 0.2

* C_ign_: cotton ignition.
